# The Novel Application of Non-Lethal Citizen Science Tissue Sampling in Recreational Fisheries

**DOI:** 10.1371/journal.pone.0135743

**Published:** 2015-09-16

**Authors:** Samuel M. Williams, Bonnie J. Holmes, Julian G. Pepperell

**Affiliations:** 1 School of Biomedical Sciences, The University of Queensland, St Lucia, Australia; 2 School of Biological Sciences, The University of Queensland, St Lucia, Australia; 3 Pepperell Research and Consulting Pty Ltd, PO Box 1475, Noosaville BC, Australia; Australian Institute of Marine Science, AUSTRALIA

## Abstract

Increasing fishing pressure and uncertainty surrounding recreational fishing catch and effort data promoted the development of alternative methods for conducting fisheries research. A pilot investigation was undertaken to engage the Australian game fishing community and promote the non-lethal collection of tissue samples from the black marlin *Istiompax indica*, a valuable recreational-only species in Australian waters, for the purpose of future genetic research. Recruitment of recreational anglers was achieved by publicizing the project in magazines, local newspapers, social media, blogs, websites and direct communication workshops at game fishing tournaments. The Game Fishing Association of Australia and the Queensland Game Fishing Association were also engaged to advertise the project and recruit participants with a focus on those anglers already involved in the tag-and-release of marlin. Participants of the program took small tissue samples using non-lethal methods which were stored for future genetic analysis. The program resulted in 165 samples from 49 participants across the known distribution of *I*. *indica* within Australian waters which was a sufficient number to facilitate a downstream population genetic analysis. The project demonstrated the potential for the development of citizen science sampling programs to collect tissue samples using non-lethal methods in order to achieve targeted research objects in recreationally caught species.

## Introduction

With growing pressure on fish stocks and the uncertainty surrounding recreational fishing catch and effort data, the application of community-based monitoring is becoming an effective tool for fishery managers [1]. Fisheries research to assess the genetic stock structure of fish communities have predominantly been applied to commercially-caught species [2], as tissue samples are easily acquired from landed fish. However, in recreational fisheries, obtaining suitable sample sizes poses a significant challenge, particularly for species that are classed as ‘no-take’ in the commercial fishing sector. To address both the need for population genetic indices for recreational-only caught species, and ameliorate the high sampling costs for genetic investigations [3,4], researchers must now explore alternative sample acquisition strategies. The use of citizen science for collection of samples by the recreational fishing community is a technique that is still in its infancy with quality control and inability to maintain interest among participants commonly hampering progress [5,6].

Technological advances have been touted as ‘the future of citizen science’ [7] by providing simplified collection methods which benefit projects through decreasing costs [8], reductions in collection errors, and increases in observational data [9]. This enhancement is largely driven by the collection of observational data using smart phones (i.e. GPS reference points and digital images) by ecological monitoring programs, many of which lack targeted research objectives [8,10]. As a result, little recent attention has been given to developing the application of direct biological sampling methods through training or recruitment of skilled volunteers to address targeted research objectives. ‘Sport’ and/or ‘game’ fishers represent a community group more broadly defined as recreational fishers who support an extensive social and geographic network of skilled and informed anglers. Engaging recreational anglers to participate in citizen science projects has been shown to deliver benefits such as acquiring samples in remote locations across broad geographic ranges that are otherwise inaccessible due to logistic and financial limitation [11] and reducing bias associated with sampling of the commercial sector [12].

Fisheries research is a discipline which has had past success in addressing targeted research objectives by employing recreational anglers to assist in the collection of biological samples (fish caught for consumption or sport) for studies on the age and growth [12–14], physiology [15,16] or reproduction [17]. However, the sampling methods used by volunteer anglers to collect biological samples remains unchanged and restricted exclusively to lethal sampling [18]. With an increasing number of fish species becoming tag-and-release or threatened, the need for developing non-lethal sampling techniques has been accentuated [18,19]. If correctly employed, the recruitment of organised and skilled anglers for research projects may potentially reduce conventional issues with quality control and participation rates, providing the opportunity for researchers to focus on developing non-lethal sampling techniques.

In Australia, the involvement of recreational anglers as citizen scientists has a rich history, with game fishers from both the private and charter sectors participating in the tag-and-release of pelagic fishes to gather new biological information as part of the fishing experience [20]. The New South Wales Game Fish Tagging Program (NSWGFTP), administered by New South Wales Department of Primary Industries (NSWDPI), has been collecting tag-and-release and recapture data on pelagic fishes continuously since 1973. This national program has been effective in collecting information on movement, growth and relative abundance of billfish, tunas, sharks and other large pelagic species within the Australian Fishing Zone and beyond [21,22]. This program operates in conjunction with the Game Fishing Association of Australia (GFAA) which encompasses 80 clubs with membership of over 8,000 registered anglers participating in gamefish tagging throughout Australia. With the support of peak recreational fishing bodies, researchers can now access recreational anglers who have previous experience in citizen science research (i.e. tagging and tournament monitoring programs) for recruitment into new sampling initiatives [23]. Recruiting skilled anglers not only increases the likelihood of participation, but also facilitates targeted research objectives, which can further enhance the range of meaningful scientific outcomes associated with the NSWGFTP.

The black marlin *Istiompax indica*, is a key focus species of the NSWGFTP, especially since it’s declaration as a commercial ‘no-take’ species in Australian waters under the *Fisheries Management Act 1991*. The large size of *I*. *indica* not only adds to its recreational appeal, but enables it to range widely throughout tropical and sub-tropical waters of the Indian and Pacific Oceans [24]. However, while the black marlin is protected from commercial fishing within the Australian Exclusive Economic Zone, its commercial catches in international waters continue to increase, particularly in the Indian Ocean where levels of fishing mortality may be to be unsustainable [25]. In addition, because of the recreational-only nature of this fishery within Australian waters, where over 90% of black marlin brought to the boat are tagged and released [23], the ability to access tissue samples from landed fish for population genetic analysis of *I*. *indica* has been limited. Given the difficulties in obtaining biological samples and the large network of skilled anglers operating under the NSWGFTP, the black marlin represents a model species for implementing citizen science research with the application of non-lethal tissue sampling, a technique that has previously been practiced exclusively by trained research scientists [26–28].

The aim of this study was to assess whether volunteer recreational anglers can collect a sufficient number of tissue samples from *I*. *indica* for future population genetic research by: (1) implementing a novel angler participation program to collect fin clips using a non-lethal sampling technique; and (2) obtain sample sizes that are sufficient for delineating population structure in future genetic research. It is hoped that the program framework described here will be used as a template for citizen science sampling of other recreationally targeted fishes to promote non-lethal sampling in the future.

## Methods

### Program implementation

From November 2012 the proposed *I*. *indica* sampling program was advertised in fishing magazines, local newspapers, social media, blogs, websites and direct communication workshops at game fishing tournaments in order to engage anglers from a range of demographic backgrounds. The most extensive communication strategy was achieved through placement of editorial content in a prominent game fishing magazine, *Bluewater Boats and Sportfishing*, with a bimonthly circulation of ~11,000 copies. In addition, social media platforms allowing real-time updates on the sampling program and preliminary results through newsletters, email lists and community fishing blogs were also utilised to engage anglers throughout Australia. The social media updates were directly linked to an external project webpage as well as other sites such as the GFAA webpage and fishing blogs where anglers could track the program’s progress.

Anglers were recruited into the program by either contacting members of our project team after being engaged by the project advertising; being contacted by a member of our team regarding their interest in participating; or becoming involved through a pre-recruited member or club. Once recruited, angler details were entered into a database which was used to maintain a mailing list for the project newsletter, provide the postage details for distributing sampling kits, and record the number of fin clips collected by each angler. The e-newsletter, *Black Marlin Bulletin*, was used as a tool to maintain regular contact with participating anglers, which was distributed via email and social media outlets. The newsletter contained a short feature article that updated anglers on recent billfish research, and reported the number of samples collected by each angler, state and club. In addition, the newsletter had the purpose of recording entries into a lucky chance draw. This competition was established to provide additional incentive for angler participation, whereby every fin clip provided gained an entry into the prize draws (two author-signed books on pelagic fish), which were drawn at the cessation of the sampling program.

### Angler sampling

Anglers recruited to the sampling program were all provided with basic sampling kits constituting one vial of 20% DMSO solution [29], a waterproof information card, zip-lock storage bags, a material safety data sheet outlining the hazards and handling techniques associated with the preservative solution, and a flyer describing the sampling methodology. Non-lethal tissue collection was undertaken by removing a ~1cm^2^ section from the dorsal or pectoral fin using rigging shears or a conventional hole punch (S1 File). The sample acquisition tool was then cleaned with fresh water to ensure no traces of blood or other material remained. Samples were placed in the 20% DMSO solution and forwarded to the senior researcher for processing. The collection of black marlin tissue samples for this study was approved by the University of Queensland animal ethics committee (AEC) for native and exotic wildlife and marine animals, Australia. To facilitate accurate reporting, biological details that anglers recorded for each sample were simplified to capture date and location and estimated mass (kg) of the fish. In the laboratory, identification of the species of every sample was independently undertaken using the microsatellite genotyping methods described in Williams *et al*. (2014) [30].

### Data analysis

An analysis of statistical power was undertaken using POWSIM to evaluate whether the number of samples collected per state were sufficient to detect genetic differentiation among populations [31]. The number of samples collected per state were simulated using previously generated pilot allele frequency data for black marlin across 18 loci by Williams *et al*., (2014) [30]. The sample sizes were evaluated as to their ability to detect significant differences (<0.05) across three levels of genetic differentiation (Fst = 0.01, 0.005 and 0.0025) using Chi-square and Fisher’s probabilities [32]. To investigate the relationship between sample size and number of participants a Pearsons correlation coefficient (r) was applied in a bivariate correlation using SAS/STAT software [33].

## Results

### Biological sampling

**Fig 1:**
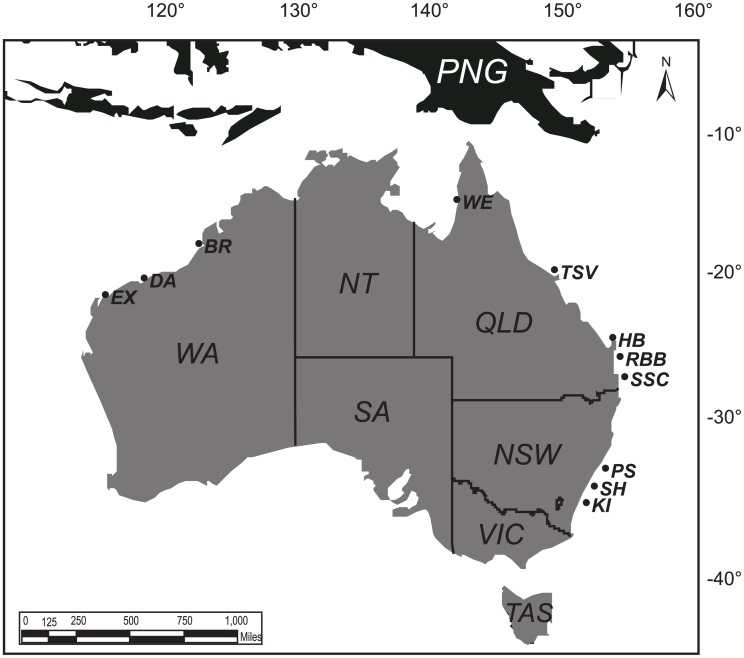
Sampling locations for *Istiompax Indica* tissue are detailed by state. PNG, Papua New Guinea; PS, Port Stephens; SH, Shell Harbour; KI, Kiama; WE, Weipa; TSV, Townsville; HB, Hervey Bay; RB, Rainbow Beach; SSC, Sunshine Coast; BR, Broome; DA, Dampier; EX, Exmouth.

Between December 2012 and November 2014, recreational anglers had provided 154 tissue samples of marlin caught and released from 13 different locations (Fig 1). All samples were subsequently genetically identified as black marlin and no cross-contamination among samples was present. Within each region the total number of samples was generally dominated by a small group of anglers, as demonstrated through the absence of a linear correlation between the number of participants and number samples collected, per location (r = 0.37). The most fin clips were received from locations in Queensland (*n* = 84), with 79% off the east coast (Sunshine Coast, n = 37; Townsville, n = 21; Hervey Bay, n = 8; Rainbow Beach, n = 1) and the remaining 21% from Weipa (n = 17) in the Gulf of Carpentaria (Table 1). In addition, 58 fin clips were received from Western Australia (Broome, *n* = 20; Dampier, *n* = 13; Exmouth, *n* = 25), 11 samples from New South Wales (Port Stephens, *n* = 9; Shell harbour, *n* = 1; Kiama, *n* = 1) and 1 sample was received from Papua New Guinea.

**Table 1:** The number of participating anglers and black marlin tissue samples collected along with the estimated overall size range from each location.

Location	Latitude (°S)	State	No. anglers	Samples (*n*)	Size range (kg)
Papua New Guinea	7	Int.	1	1	20
Port Stephens	32	NSW	3	9	11–50
Shell harbour	34	NSW	1	1	55
Kiama	34	NSW	1	1	78
Weipa	12	QLD	8	17	8–20
Townsville	19	QLD	6	21	8–25
Hervey Bay	24	QLD	5	8	3–30
Rainbow Beach	25	QLD	1	1	2.5
Sunshine Coast	26	QLD	5	37	5–25
Broome	17	WA	2	20	4–65
Dampier	20	WA	9	13	20–50
Exmouth	21	WA	9	25	9–80

### Power analysis

A power simulation was undertaken to investigate whether the sample sizes collected were sufficient to permit the identification of genetic differentiation (QLD = 84, WA = 58 and NSW = 11). The power analysis suggested that these sample sizes were sufficient to recognise population subdivision in 100% of runs at the highest level of genetic differentiation (Fst = 0.01) for both the Chi-Squared and Fishers test. When the level of differentiation was decreased to 0.005 the sample sizes were still sufficient to detect differences in ~93% of runs across both tests (Chi-Square = 93.6, Fishers = 92.60). When the power analysis was conducted at an Fst of 0.0025, significant differentiation amongst population was only estimated in 57.4% and 52.60% of runs by the Chi-Square and Fishers tests, respectively.

## Discussion

Though some previous contributions of recreational anglers have been able to facilitate research that has produced significant insights into the understanding of marine species [12,17], the lack of targeted scientific objectives have largely restricted further application of this underutilised resource [1,34,35]. The non-lethal collection of fin clips from *I*. *indica* by recreational anglers demonstrated here represents an advancement in citizen science fisheries research. By accessing a group of participants pre-involved in citizen science activities (tagging, tournament monitoring), the project was able to gain high participation rates over a short timeframe. The success of this program can be demonstrated through the sample sizes collected in this study constituting a sufficient number to permit future statistically valid population genetic research on *I*. *indica*. The communication of regular feedback to anglers was, without doubt, instrumental in achieving these required samples and fundamental in maintaining high participation rates. The effective development and implementation of this program not only offered an opportunity for recreational fishing communities to become involved in scientific research, but takes a step forward in the collection of biological data through citizen science.

It is not a coincidence that successful citizen science programs often mention feedback as a key means of maintaining interest in volunteering [36,37], with much of the success of maintaining participation in this project able to be attributed to feedback provided via social media and e-newsletters. Providing regular feedback of information to the fishery was essential in consolidating the working relationship between fishers and project scientists. It also provided the opportunity for anglers to suggest ways of improving future non-lethal sampling protocols. An example of angler feedback was the suggestion that using biopsy needles to simultaneously tag and remove small tissue samples from fish with a tagging pole, similar to those methods used in Buckworth *et al*., (2012) [38], may increase overall sample numbers. It was proposed that by employing this technique it would reduce safety concerns associated with handling of highly-energetic fish at the side of the boat, by placing a greater distance between an angler and the captured fish.

A number of other elements also made this citizen science program particularly successful. Firstly, the black marlin is a prime tag-and-release species in Australia, with over 58,000 fish having been tagged with conventional tags to date by recreational anglers, the highest for any species on the NSW Game Fish Tagging Program [23]. This familiarity with the species negated any misidentification, with 100% of samples proven genetically to be black marlin. Secondly, the prior experience in citizen science activities by program participants also meant that the amount of angler education regarding species identification, data management, and fish handling skills was able to be minimised, with focus able to be put on sampling methodology, preservation of the genetic material and returning samples to researchers.

Limitations regarding the sampling program were also identified. One such limitation was the overall sample collection being largely dominated by a small group of enthusiastic anglers. This sampling bias was statistically reflected through a weak correlation between the number of samples collected and number of participants that would be expected if all participants contributed equally (r = 0.37). Although numbers of hours fished per sample were unquantified in this study, the contribution of samples to the program reflected the number of fishing trips by participating anglers. This is best demonstrated by recreational charter operators contributing considerably more samples than ‘sports’ focused recreational anglers fishing from their own vessels. The seasonal absence of fish in some regions due to unfavourable environmental conditions during the sampling period also presented a geographic limitation. This meant that despite angler participation and enthusiasm, no samples were able to be collected from some areas. In light of this, it should be noted that the sampling pattern outlined here only represents those regions and anglers that collected samples, not those who participated, but were unable to collect samples. These limitations highlight the necessity for careful planning and execution of future citizen science sampling programs, and that special consideration should be given to sampling design to minimise bias and supply sufficient sample sizes where necessary to achieve the targeted research objectives.

The success of the tissue sampling program demonstrates that citizen science can be utilised for non-lethal sampling of recreationally-caught fish. Given that researchers have applied non-lethal sampling for studies on such factors as diet [39], heavy metal contamination [28] and reproductive biology [40], the success of this program promotes the possibility of citizen scientists assisting with the collection of biological samples for future research in the aforementioned fields. It is hoped that by defining the contribution of biological samples by recreational anglers as ‘citizen science’, it will also encourage future research projects to regonise similar contributions in the same way. The approach detailed in this paper recognises that citizen science can advance in non-technological forms through biological sampling techniques, providing further incentive for researchers to explore alternative non-lethal sampling strategies to further the biological understanding of their study species.

## Supporting Information

S1 File An angler demonstrating how to take a fin clip from a juvenile black marlin off Weipa (Queensland). (MOV)
